# The Th17 Pathway in Vascular Inflammation: Culprit or Consort?

**DOI:** 10.3389/fimmu.2022.888763

**Published:** 2022-04-11

**Authors:** Marie Robert, Pierre Miossec, Arnaud Hot

**Affiliations:** ^1^ Department of Clinical Immunology and Rheumatology, and Immunogenomics and Inflammation Research Unit, University of Lyon, Hôpital Edouard Herriot, Lyon, France; ^2^ Department of Internal Medicine, University of Lyon, Hôpital Edouard Herriot, Lyon, France

**Keywords:** interleukin-17, Th17 cells, cardiovascular system, allo-immune vascular inflammation, vasculitis, IL-17 inhibitors

## Abstract

The involvement of IL-17A in autoimmune and inflammatory diseases has prompted the development of therapeutic strategies to block the Th17 pathway. Promising results came from their use in psoriasis and in ankylosing spondylitis. IL-17A acts on various cell types and has both local and systemic effects. Considering the premature mortality observed during chronic inflammatory diseases, IL-17A action on vascular cells was studied. Both *in vitro* and *in vivo* results suggest that this cytokine favors inflammation, coagulation and thrombosis and promotes the occurrence of cardiovascular events. These observations led to study the role of IL-17A in diseases characterized by vascular inflammation, namely allograft rejection and vasculitis. Increased circulating levels of IL-17A and histological staining reveal that the Th17 pathway is involved in the pathogenesis of these diseases. Vasculitis treatment faces challenges while the use of steroids has many side effects. Regarding results obtained in giant cell arteritis with IL-6 inhibitors, a cytokine involved in Th17 differentiation, the use of anti-IL-17 is a promising strategy. However, lessons from rheumatoid arthritis and multiple sclerosis must be learnt before targeting IL-17 in vasculitis, which may be culprit, consort or both of them.

## 1 Introduction

Interleukin (IL)-17A is a pro-inflammatory cytokine involved in many autoimmune and inflammatory diseases (1). Its identification in the pathogenesis of these disorders had led to the development of therapeutics with a great success in psoriasis and in ankylosing spondylitis (2, 3). Outside its local effects, IL-17A induces systemic manifestations playing a role in the premature cardiovascular (CV) mortality observed in inflammatory diseases (4–6). IL-17A acts on all cell types that make up the three layers of the vascular wall by promoting inflammation, coagulation and thrombosis (7). These results suggest that IL-17A is involved in vascular inflammation and particularly in allograft rejection and vasculitis.

Vasculitis are defined according to the size and the type of the vessels that are predominantly affected (8, 9). From large to small vasculitis, histopathological lesions are different but evidence hints that T-helper (Th)-17 cells and IL-17A are involved in their pathogenesis (1). In many cases, the treatment relies on steroids which have short and long-term side effects and alternative therapeutics are expected (10–13). Given the role of the Th17 pathway in these diseases, one can expect that the inhibition of IL-17A could be of a great interest. Indirect evidence came from the blockade of IL-6, a cytokine required for Th17 differentiation, that is recommended in selected cases of large vessel vasculitis (LVV) (14, 15). However, disappointing use of IL-17 inhibitors in rheumatoid arthritis (RA) and in multiple sclerosis, while the Th17 pathway is clearly involved in the pathogenesis, must be understood before going further into their application in vasculitis (16, 17).

In this review, the key effects of the Th17 pathway on blood vessels will be detailed, after a brief overview of the Th17 pathway. Then, its involvement in vasculitis will be addressed. Therapeutic implications are finally discussed.

## 2 Effects of IL-17 on Blood Vessels

### 2.1 Overview of the Th17 Pathway

#### 2.1.1 IL-17 Cytokines

Six isoforms (IL-17A to IL-17F) compose the IL-17 family (18–23). IL-17A has pleiotropic effects with a key role in host defense against extracellular pathogens, including bacteria and fungi, but also in chronic inflammation and autoimmunity (1, 16). IL-17A and IL-17F bear the greatest homology and are secreted either as homodimer or heterodimer (23, 24). Both IL-17A and IL-17F drive inflammation, IL-17F being less potent than IL-17A (25). In the presence of tumor necrosis factor (TNF)-α, they induce rather similar expression profiles (26, 27).

IL-17E (or IL-25) has the lowest homology with IL-17A and promotes Th2-cell mediated immune responses (28). Infection of the lungs with an IL-25 expressing adenovirus or IL-17E protein induces IL-4, IL-5, IL-13 production and then eosinophil infiltration, mucus secretion and airway hyperreactivity (29). IL-17E axis plays a role in asthma exacerbations and now constitutes an attractive target for the development of new therapies (30). IL-17E also modulates Th17 cell function by acting as a receptor antagonist for IL-17A function (31–33).

#### 2.1.2 IL-17 Receptor Family and Signaling

Five receptors (IL-17RA to IL-17RE) compose the IL-17 receptor (IL-17R) family (32). IL-17RA interacts with other subunits to form receptor complexes. IL-17A, IL-17F or IL-17A/F signal through IL-17RA/RC. IL-17E binds to IL-17RA/RB and IL-17C to IL-17RA/RE (28). IL-17RD is an alternate receptor subunit for IL-17A but not for IL-17F (34).

IL-17 signaling activates nuclear factor kappa B (NFκB), CCAAT/enhancer binding protein (CEBP)-ß/δ and mitogen-activated protein kinase pathways. It activates inflammatory genes encoding cytokines and chemokines (e.g., IL-6 and IL-8). IL-17 can also regulate genes post-transcriptionally and mRNA half-life (34).

IL-17 function is regulated by different mediators. TNFα, IL-1, granulocyte-macrophage colony stimulating factor (GM-CSF) and interferon (IFN) γ regulate positively IL-17 effects whereas IL-17E/IL-25, anti-IL-17 auto-antibodies and soluble IL-17R inhibit its function (17).

#### 2.1.3 IL-17 Producing Cells

Th17 cells undergo differentiation following three steps. The first step corresponds to the initiation of the differentiation and is mediated by transforming growth factor (TGF)-ß and IL-21. Both cytokines induce the transcription of the lineage specific transcription factor receptor-related orphan receptor (RORc). Secondly, IL-6 and IL-1ß allow the amplification of the Th17 lineage. Finally, Th17 cells acquire their pathogenic role thanks to IL-23. Th17 produce many cytokines as IL-17A, IL-17F, IL-21 and IL-22 (15, 35).

A balance exists between Th17 and Treg cells because their developmental pathways are interconnected and reciprocally regulated. TGF-ß is necessary for both Th17 and Treg differentiation. The addition of IL-6 inhibits FoxP3, required for Treg differentiation, and upregulates RORc. This induces a shift toward the Th17 lineage (15). Moreover, mice deficient in exons 2 and 7 of FoxP3 fail to repress RORc, have increased levels of Th1 and Th17 cytokines and exhibit multi-organ inflammation with Treg lacking their suppressive ability (36). In humans, a substantial number of inflammatory diseases are characterized by an increase of Treg lacking exon 2 of FoxP3, with Treg unable to control IL-17^+^ T cell proliferation. These Δexon 2 FoxP3 Tregs but also some Tregs in inflammatory conditions can also produce IL-17. These results highlight the plasticity between these two cell types (37–39).

Overall, the Th17/Treg balance plays a key role in autoimmune and inflammatory diseases; Treg cells prevent their development while Th17 cells promote them (15). These dynamic changes illustrate the importance of the cytokine environment but are also influenced by local interactions (35).

Other IL-17 sources include immune cells with γδ T cells, invariant natural killer cells, innate lymphoid cells, CD8^+^ T cells or double-negative T cells (28, 40). Mast cells and neutrophils do not produce IL-17 but engulf it (41, 42).

**Figure 1:**
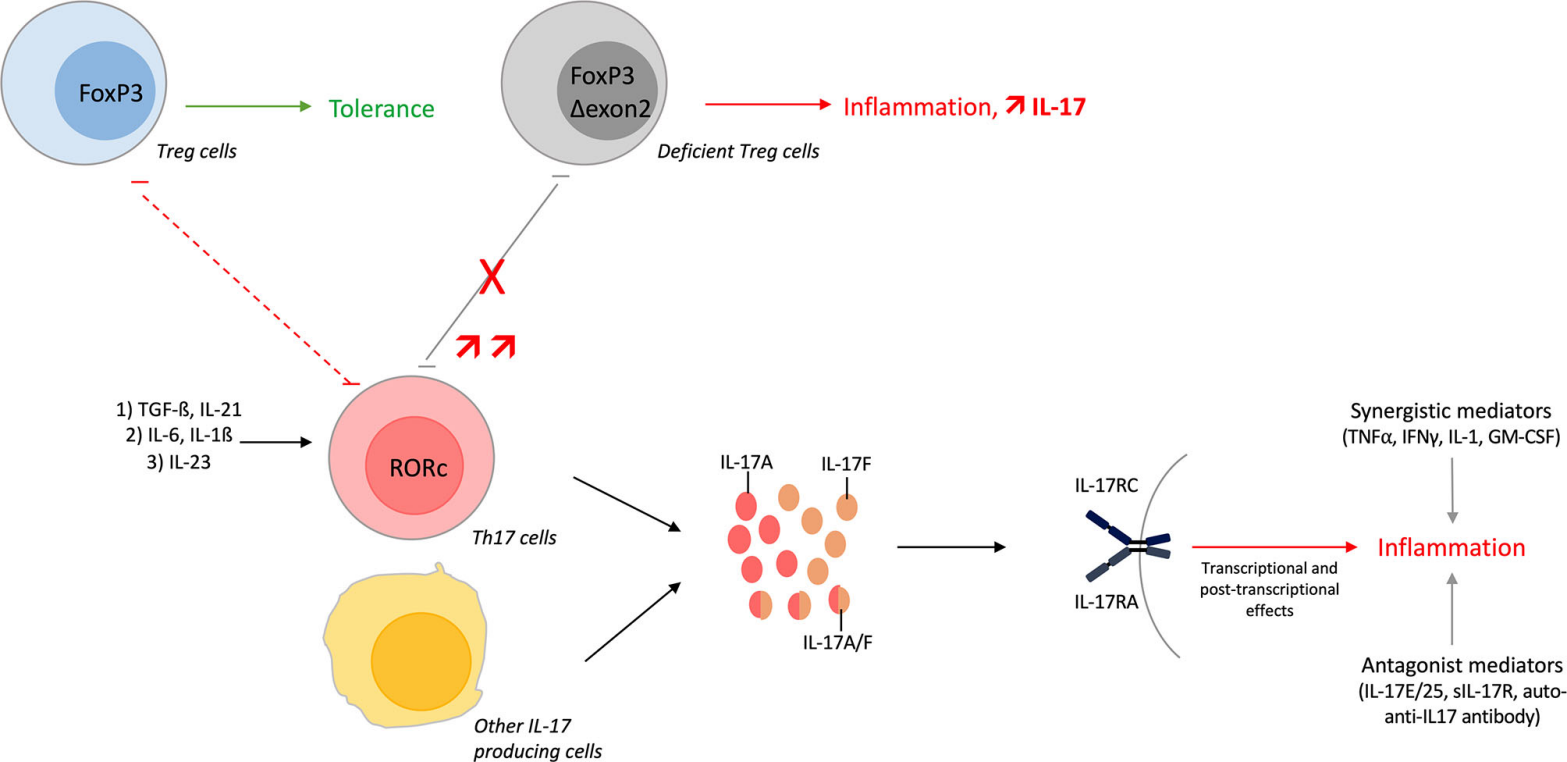
Overview of the Th17 pathway. T-helper (Th) 17 cells undergo differentiation from Th0 cells. The first step involves transforming growth factor (TGF)-ß and interleukin (IL)-21 that initiate the differentiation. Next, IL-6 and IL-1ß amplify the Th17 lineage and finally, IL-23 is required to maintain the lineage and to acquire its pathogenic role. Th17 differentiation can be shifted towards regulatory T (Treg) cells depending on cytokine environment. Developmental pathways of both cells are interconnected and reciprocally inhibited. Treg lacking exon 2 of FoxP3 impairs the Th17/Treg balance with increase amount of IL-17^+^ T cells. These Δexon 2 FoxP3 Treg cells, and some Tregs in inflammatory conditions, can also produce IL-17. Moreover, IL-17A/F/AF are produced by Th17 cells but also by other immune cells. These cytokines bind the same receptor, activate different pathways and finally induce inflammation. IL-17 function is regulated positively or negatively by different mediators.

These elements are summarized in **Figure 1**.

### 2.2 Results on Isolated Cells

**Figure 2:**
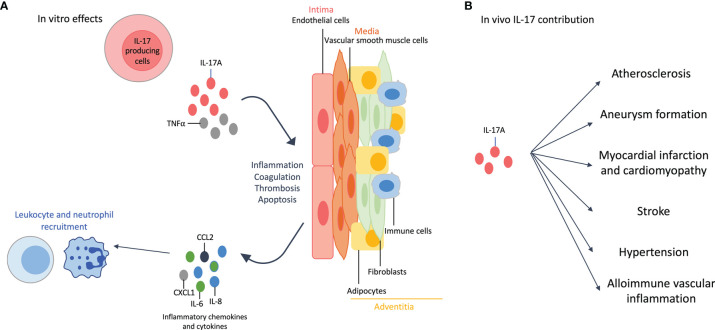
Effects of interleukin (IL)-17A on the cardiovascular system. **(A)**: *In vitro* effects: IL-17A, and even more when combined with tumor necrosis factor (TNF)-α, affects all cell types of the vascular wall. IL-17A +/- TNFα induces inflammation with the release of pro-inflammatory cytokines (e.g., IL-6) and chemokines (e.g., IL-8, chemokine C-X-C motif ligand 1 CXCL1, C-C Motif Chemokine Ligand 2 CCL2), that in turn enhances neutrophil and leukocyte recruitment. This recruitment is also favored by the increased expression of adhesion molecules. IL-17A induces thrombosis, coagulation and apoptosis. The effects on adipocytes participate to the inflammatory environment. **(B)**: *In vivo* contribution of IL-17A results in accelerated atherosclerosis, aneurysm formation, myocardial infarction and cardiomyopathy, stroke, hypertension and allo-immune vascular inflammation.

The vascular wall is composed of three layers: the intima, the media and the adventitia. Briefly, endothelial cells (EC) are part of the intima, vascular smooth muscle cells (VSMC) of the media and adipocytes, fibroblasts and immune cells of the adventitia. IL-17A alone, and even more when combined with TNFα, acts on these cell types (**Figure 2A**).

#### 2.2.1 Effects on Intima Cells

IL-17A induces the secretion of pro-inflammatory cytokines (e.g., IL-6) and chemokines (e.g., IL-8, chemokine (C-X-C motif) ligand 1 CXCL1, C-C Motif Chemokine Ligand 2 CCL2) by EC (43). It also increases the level of adhesion molecules, especially when combined with TNFα, and then promotes leukocyte recruitment and invasion of EC (7). IL-17A promotes thrombosis and coagulation by activating tissue factor and reducing anti-coagulation mediators (e.g., CD39 and thrombomodulin) (7, 44). Finally, IL-17A increases EC apoptosis (45).

#### 2.2.2 Effects on Media Cells

VSMC play a key role in atherosclerosis through their ability of proliferation, migration and apoptosis. IL-17A increases the production of pro-inflammatory cytokines and chemokines and the expression of adhesion molecules, plaque destabilizing proteins and tissue factor. VSMC apoptosis is also increased by IL-17A when combined with TNFα and/or IFNγ, that in turn promotes atherosclerosis (45, 46).

#### 2.2.3 Effects on Adventitia Cells

IL-17A triggers inflammation through the production of pro-inflammatory cytokines by the different cell types of the adventitia but also by enhancing adipocyte lipolysis (7, 47).

IL-17 effects on isolated cells are summarized in **Figure 2A**.

### 2.3 Results From *In Vivo* Experiments and Systemic Effects of IL-17A

#### 2.3.1 Effects of IL-17 on the CV System *In Vivo*

IL-17A, alone or combined with IFNγ, increases lesion size and plaque instability by inducing inflammation and enhancing the recruitment of myeloid cells. Other results show that IL-17A favors aneurysm formation, myocardial infarction, stroke and hypertension. However, some results are contradictory regarding the protective or the deleterious effect of IL-17 on CV outcome. This is particularly true for atherosclerosis where animal models and protocols used are different across experiments (7).

#### 2.3.2 Allo-Immune Vascular Rejection as a Paradigm of IL-17 Vascular Pathology

Blood vessels in allotransplantation remain largely of graft origin and are subject to host allo-immune responses. Vascular pathology can contribute to graft inflammation, ischemia/reperfusion injury and then allograft rejection. Mediators of innate and adaptive immunity are involved in these processes and support both hyper-acute, acute and chronic rejections (48). Among adaptive immune cells, the Th17 subset contributes to allograft rejection (49).

During acute rejection, IL-17 blockade significantly improves cardiac graft survival in a rat model (50). The antagonism of IL-17 decreases mononuclear infiltration and endothelial damage in a murine aortic transplantation model (51). Allograft rejection, partially mediated by IL-17, mainly relies on neutrophil recruitment (49, 52).

Chronic rejection is made up of a parenchymal and a vascular rejection. The latter is caused by a stenosis of vessels due to a progressive immune mediated host response to graft blood vessels. Different mechanisms contribute to stenosis including ischemia/reperfusion, hypertension and immune activation (53). IL-17 and Th17 cells play a role in these phenomenon (17). A model of chronic allograft vasculopathy shows that, in absence of Th1 response, Th17 cells induce severe accelerated allograft rejection and vasculopathy (54). Reducing IL-17 production suppresses chronic allograft rejection and vasculopathy (55). IL-17^-/-^ mice do not develop cardiac fibrosis after mismatched organ transfer, which is considered as secondary to allograft vasculopathy (56). Also, in IL-17 deficient mice, graft coronary artery disease after heterotopic cardiac transplantations is reduced (57).

#### 2.3.3 Circulating Levels of IL-17 in Human Vascular Pathology

In addition to its local effects, IL-17A is circulating and has systemic effects (4). As mentioned above, IL-17A function is regulated by various mediators (17). To counteract these complex interactions, a bioassay was developed to study specifically the bioactive fraction of IL-17A (58). This was particularly well described in rheumatoid patients where bioactive IL-17A is associated with joint destruction and CVE occurrence (5, 59). Similarly, patients with myocardial infarction show a peak of circulating IL-17A at admission (60). IL-17A induces systemic effects by affecting various cell types: cells of the liver, of skeletal and cardiac muscles and of the blood vessels (4).

*In vivo* contribution of the Th17 lineage is described in **Figure 2B**.

## 3 Role of the Th17 Pathway in Vasculitis

Regarding IL-17A effects on the CV system, this cytokine could play a role in vasculitis pathogenesis which is characterized by blood vessel wall inflammation, endothelial injury and tissue damage.

Only noninfectious vasculitis of the 2012 International Chapel Hill Consensus Conference (CHCC2012), partially revised in 2018, are described (8, 9). Vasculitis are classified according to the size and the type of vessels predominantly affected. The effects of IL-17 and Th17 in large vessel vasculitis (LVV), medium vessel vasculitis (MVV), small vasculitis (SVV) and variable vessel vasculitis (VVV) are addressed.

### 3.1 Large Vessel Vasculitis (LVV)

**Figure 3:**
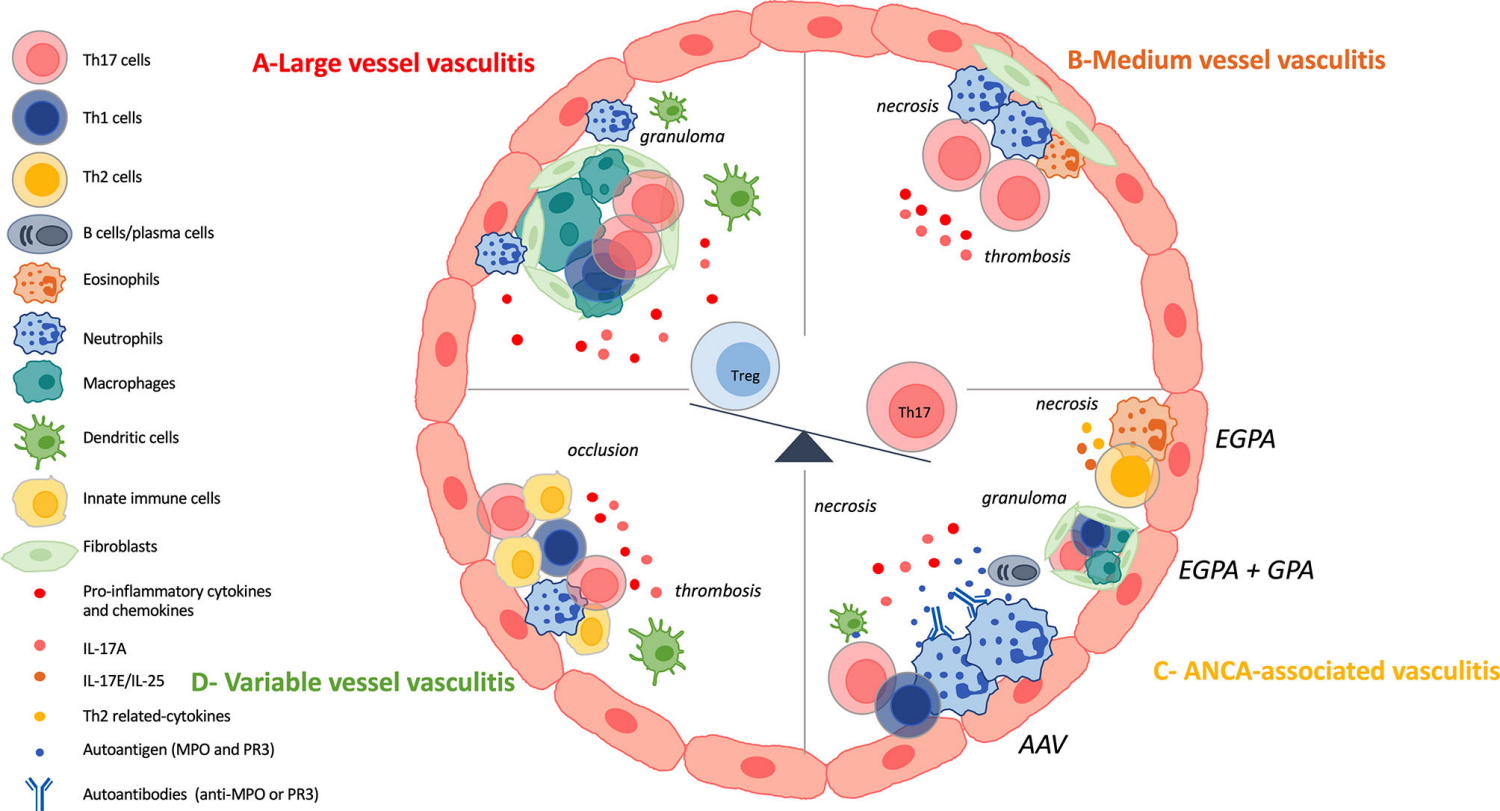
IL-17 and Th17 involvement in vasculitis. **(A)**: Large vessel vasculitis (LVV) include giant cell arteritis (GCA) and Takayasu arteritis (TAK) and are characterized by a chronic granulomatous inflammatory reaction. Vascular adventitial dendritic cells are activated and induce the recruitment, the activation and the polarization of CD4^+^ T cells. Among CD4^+^ T cells, T-helper (Th)-17 cells and Th1 cells are involved in the local inflammatory process. Then, CD8^+^T cells and monocytes are recruited and finally, vascular remodeling occurs. Th17 cells are also circulating and patients harbor increased frequency of them. IL-17A promotes neutrophil recruitment. There is a decrease of regulatory T (Treg) cells at the expense of the Th17 lineage. **(B)**: Medium vessel vasculitis (MVV) comprise polyarteritis nodosa (PAN) and Kawasaki disease (KD). There are few data on Th17 involvement in PAN pathogenesis but patients harbor defective Tregs and increased frequency of Th17 cells. KD is characterized by necrotizing arteritis with neutrophil infiltration. Lymphocytes, eosinophils and myofibroblastic proliferation participate in KD arteriopathy. Th17 cells and their related cytokines are increased in KD and there is a dysregulation of the Th17/Treg balance. **(C)**: Antineutrophil cytoplasmic antibody (ANCA)-associated vasculitis (AAV) are composed of three variants: microscopic polyangiitis (MPA), granulomatosis with polyangiitis (GPA), eosinophilic granulomatosis with polyangiitis (EGPA). They are characterized by fibrinoid necrosis, thrombus formation and immune cell infiltration. GPA and EGPA are associated with granulomatous inflammatory reaction where Th17, Th1 cells and macrophages play a role. EGPA is characterized by the involvement of eosinophils, Th2 cells and their related cytokines. GPA and MPA, and sometimes EGPA, are associated with ANCA. There is a loss of B and T tolerance toward MPO and PR3 antigens, that are found primary in neutrophils. PR3 and MPO-specific Th17 responses occur in the course of the disease. IL-17A also favors the recruitment of neutrophils at the site of injury. There is a dysregulated Th17/Treg cells balance. **(D)**: Variable vessel vasculitis (VVV) can affect any type and any size of vessels. Enhanced innate immune response, early tissue infiltration and late adaptive immunity are the different steps that occurred in Behçet’s disease (BD) pathogenesis. This results in thrombosis, occlusion and aneurysms. Th17 cells are found at the site of tissue injury and interact with other immune cells. There is an hyperactivation of Th17 and Th1 cells at the expense of Treg population.

LVV mainly affect large arteries including the aorta and its major branches. The two major diseases are Takayasu arteritis (TAK) and giant-cell arteritis (GCA) (8). Both disorders occur mainly in females and share histopathologic features with a chronic granulomatous inflammatory reaction (61). They differ by the age of onset: TAK generally occurs before the age of 50 years old whereas GCA after age 50 (62). Chronic inflammation within the vessel wall can lead to aneurysm formation, rupture or dissection where IL-17A and Th17 cells play a role (7, 63). Th17 cells are identifiable both in the peripheral blood and in the vasculitic lesions. It raises the possibility that inflammatory cells recirculate (63). **Figure 3A** summarizes the results described below.

#### 3.1.1 Takayasu Arteritis (TAK)

TAK is a rare disease that induces acute inflammation, carotidynia, discrepant blood pressure between the arms, absent or asymmetric pulsation, limb claudication and angina (64).

Patients with TAK have significantly increased level of IL-17A and circulating Th17 cells compared with healthy individuals (65, 66). Similar results are obtained when comparing patients with active TAK with those in remission (66–68). After anti-CD3/CD28 stimulation, IL-17A-producing T cell frequency is significantly increased in the presence of serum from TAK patients with active disease compared with those in remission (66). Anti-endothelial protein C receptor (EPCR) antibodies, found in 34.6% of TAK patients, impair Th17 differentiation (69). The imbalance that exists between Th1/Th17 and Treg cells in TAK is driven by type I and II cytokines which signal through the Janus kinase/signal transducers and activators of transcription (JAK/STAT) pathway (70). The differentiation of Th1 and Th17 cells is impaired in TAK patients and mediated by an overactivation of mammalian target of rapamycin complex 1 (mTORC1) (71). Recently, an increase in Th17.1 cells producing both IL-17 and IFNγ was observed in TAK patients compared with healthy controls. Similar results were described for PD1^+^Th17 cells (72). Finally, IL-17A and Th17-related cytokines trigger neutrophil recruitment and activation that could contribute to vascular lesions (73).

Among Th17-related cytokines (IL-6, IL-21, IL-23, IL-1ß, TGF-ß), the role of IL-6 in TAK pathogenesis has been well described while IL-6 is critically involved in Th17 differentiation (15). Its level is increased in TAK patients compared with controls and linked to disease activity (66, 68). Similar trends are described for IL-23 level in TAK (65, 66).

#### 3.1.2 Giant-Cell Arteritis (GCA)

GCA affects older patients than TAK and typically induces vasculitis of the extracranial branches of the aorta (62, 74). The three layers of the arterial wall are affected by histopathological lesions, especially the internal elastic lamina with multinucleated giant cells, CD4^+^ T cells and macrophages organized in granuloma (74). Recent understanding of the immunopathological model of GCA has allowed to divide its pathogenesis into four steps. The first corresponds to the activation of vascular adventitial dendritic cells, the second is characterized by the recruitment, the activation, and the polarization of CD4^+^ T cells, followed by the recruitment of CD8^+^T cells and monocytes. Finally, vascular remodeling occurs. Th17 cells are mainly involved in the second phase (75, 76). Th17 pathogenic role in vascular remodeling is similar to the one described in myocardial infarction (7).

Frequencies of circulating Th17 cells are increased up to 10-fold in untreated GCA patients compared with healthy controls (77–79). Immunohistochemical analysis of temporal artery biopsy specimens from GCA patients show infiltration of Th17 cells, mainly localized at the junction between the adventitia and the media. Artery-infiltrating Th1 cells may derive from local differentiation of Th17 cells in the presence of IL-12. CD161^+^CD4^+^ T cells could be the common precursor that links Th1 and Th17 cells (76, 78). As well as Th1 cells, a population of IL-17/IFNγ double producing cells is expanded in untreated GCA patients to create granuloma and IL-17 regulates macrophage recruitment (63, 77, 78). IFNγ^+^ CD4^+^ T cell commitment involves JAK/STAT pathway and type I interferon signature is upregulated in GCA aortas (80, 81). Induction of both T-bet^+^-CD4^+^ and RORγt^+^CD4^+^ T cells involves the AKT-mTORC1 signaling pathway and there is a constitutive activation of the Notch-AKT-mTORC1 signaling axis in T cells from GCA patients (82).

An imbalance between Th17 and Treg cells is observed in GCA patients compared with healthy subjects. This balance is partially modulated by IL-6 and IL-21, whose levels are correlated with disease activity (78, 79, 83). GCA Tregs may increase vascular inflammation by promoting Th17 polarization (37).

In a model of severe combined-immunodeficiency (SCID) mice engrafted with normal human arteries, treatment with dexamethasone inhibits both mRNA production of IL-17 and Th17 density in the vessel wall (77). Similar results are observed in patients with a decrease of IL-17 producing CD4^+^ T cells after steroid therapy and the Th17/Treg ratio is significantly reduced (78). However, it does not restore the Treg deficiency observed in GCA and does not affect Th1 response (77, 78). Moreover, in a chimeric mouse model of GCA, the inhibition of JAK1/JAK3 activity reduces RORc expression and IL-17 level, and finally suppresses T-cell invasion and proliferation into the artery (80).

Results described for Th17 cells are confirmed at the cytokine level. IL-17 and Th17-related cytokines (IL-1ß, IL-6, IL-21, IL-23) levels are increased in serum from untreated GCA patients and after PBMCs stimulation with PMA/ionomycin from patients with active disease compared with controls and those in remission (77, 79). After steroid therapy, the level of circulating IL-17 is decreased (77). Similar observations apply for IL-17A, IL-1ß, IL-6 and IL-23 expression in inflamed temporal arteries (77, 79, 84, 85). IL-17 is overexpressed when transmural inflammation and granulomatous reaction occur (84).

Finally, studies of genetic background and epigenetic modifications suggest a role of IL-17 and Th17 pathway in GCA development or pathogenesis (75, 86, 87).

### 3.2 Medium Vessel Vasculitis (MVV)

Polyarteritis nodosa (PAN) and Kawasaki disease (KD) are the two entities that constitute MVV. **Figure 3B** gives a brief overview of Th17 involvement in the pathogenesis.

#### 3.2.1 Polyarteritis Nodosa (PAN)

PAN generally occurs in patients of 50 years old and can be primary or secondary to viral infection with the example of hepatitis-B-virus. It induces renal vasculitis with renovascular hypertension, renal infarcts and microaneurysms (88).

Very few studies refer to the effects of IL-17 or Th17 cells in PAN. IL-17 producing CD4^+^ T cells frequency is higher in PAN patients compared with healthy controls. PAN patients also have defective Tregs in suppressive ability (89). In PAN patients with cutaneous mutations, trends toward a decrease of IL-17 level after treatment are observed without significant difference (90).

#### 3.2.2 Kawasaki Disease (KD)

KD is the leading cause of acquired heart disease in children and primarily involves muscular arteries (91). Three pathological processes have been identified to explain KD arteriopathy: necrotizing arteritis characterized by neutrophilic infiltration, subacute and chronic vasculitis with an inflammatory cell infiltrate composed of lymphocytes, plasma cells and eosinophils and luminal myofibroblastic proliferation. These lesions can induce coronary artery aneurysm, thrombosis, stenosis and myocardial infarction (92). Systemic inflammation goes along with vascular lesions and could be mediated by inflammatory cytokines, as IL-17.

Th17 cells and related cytokines (IL-17A, IL-6, IL-23, IL-21 and IL-22) are markedly increased in KD patients compared with controls. These observations have been made both in the plasma, in the serum and in the supernatants of cultured PBMCs after stimulation with anti-CD3/CD28 (93–95). Some results suggest that IL-17 level is correlated with disease activity (93, 96). Moreover, myofibroblasts expressing IL-17 and IL-6 are observed in the damaged arterial wall of KD autopsies (97). Regarding the Th17/Treg balance, Treg frequency and FoxP3 expression are markedly lower in KD patients compared with controls suggesting a shift towards Th17 differentiation, probably mediated by IL-6 (93, 98).

Cytokine levels and Th17 frequency are decreased after one week of combined therapy including aspirin and intravenous immunoglobulin (IVIG) (94). IVIG-resistant KD have increased plasma levels of IL-17A and IL-6 before treatment compared with sensitive patients. Resistant patients maintain high levels of these cytokines after treatment (93). To counteract this resistance, plasma exchanges have been tested. It induces the removal of IL-17 and IL-6 levels and could participate in therapeutic mechanisms (99).

### 3.3 Small Vessel Vasculitis (SVV)

Small intraparenchymal arteries, arterioles, capillaries and venules are mainly affected in SVV. Two categories of SVV are described based on paucity or abundance of vessel wall immunoglobulin deposits (8).

#### 3.3.1 Antineutrophil Cytoplasmic Antibody (ANCA)-Associated Vasculitis (AAV)

Results suggest that the Th17 subset is involved in AAV pathogenesis as IL-17 serum level is increased in AAV patients compared with healthy individuals. Similar observations apply for IL-23. However, levels remain elevated in some patients and major relapses occur suggesting that a Th17 memory cell population may persist (100). The proportion CCR6^+^CD4^+^RORγt^+^ T cells is increased in kidney biopsy samples compared to the peripheral blood suggesting recruitment of these cells into the kidney. These results support the role of Th17 in AAV pathogenesis (101, 102). The conversion from Tregs to Th17 effector cells within the inflammatory environment play a role in AAV pathogenesis and the imbalance of Th17/activated Treg cells marks renal involvement (103, 104). Moreover, Treg cells harbor a defect in their suppressive function (39).

Among other IL-17 cytokines, serum IL-17C level is increased in AAV-patients with crescentic glomerulonephritis (GN) compared with controls. Results from mice models confirm that IL-17C promotes tissue renal injury in an IL-17A-dependant manner. IL-17RE promotes Th17 response in crescentic GN and is expressed on Th17 cells. The activation of the IL-17C/IL-17RE axis increases renal expression of IL-17 target genes leading to neutrophil recruitment and then tissue injury (105).

##### 3.3.1.1 Microscopic Polyangiitis (MPA)

MPA belongs to AAV and affects mainly the kidneys with progressive GN and the lungs with alveolar hemorrhage (106). MPA is mainly associated with MPO-ANCA (107). IL-17A level is increased in MPA patients compared with controls (108). Th17 and Th1 cells promote macrophage activation at sites of injury. Macrophages hasten disease progression through their profibrotic properties (109). In murine model of anti-MPO induced GN, IL-17 may enhance antigen deposition in the glomeruli and mediates pathogenic effector functions (110). Toll-like receptor (TLR)-2 ligand promotes Th17-induced MPO autoimmunity (111). MPO-specific Th17 cells are involved earlier in disease while Th1 cells are implicated later (112). CD8^+^ T cells also cause experimental injury (113).

##### 3.3.1.2 Granulomatosis With Polyangiitis (GPA)

GPA is characterized by a necrotizing granulomatous inflammation and is predominantly associated with PR3-ANCA (107). It usually involves the upper and lower respiratory tract and kidneys. Almost all patients have sinonasal involvement (114). Patients with GPA have an increased percentage of circulating IL-17A^+^T cells compared with healthy controls (115, 116). Involvement of the eye socket in GPA is rare but cytokine staining for IL-17 and IL-23 are significantly greater in GPA lesions compared with idiopathic inflammatory orbital diseases and sarcoidosis lesions (114, 117). The increased production of IL-17A could in turn enhances neutrophil recruitment and activation that are involved in AAV pathogenesis (118). Stimulation with the PR3 autoantigen increases Th17 cell frequency in ANCA-positive GPA patients compared with ANCA-negative ones and healthy individuals, suggesting the existence of PR3-specific Th17 responses (119, 120). Moreover, sustained remission is characterized by an increase of Treg, Th2 cells and Th2-cytokines levels (116, 121, 122). The increase of Treg cells is not sufficient to confer enhanced suppression (122, 123). Among FoxP3^+^ cells, there is a marked increase of non Treg cell subtype in GPA-patients in remission compared with healthy donors. These cells produce significantly more pro-inflammatory cytokines (e.g., IL-17) in ANCA-positive patients compared with ANCA-negative ones and healthy controls (124). Finally, recent results suggest an interaction between regulatory B (Breg) cells and Th17 cells. There is a negative correlation between Th17 effector memory cells and Bregs, and *in vitro* experiments show an expansion of Th17 cells after Breg depletion (125).

##### 3.3.1.3 Eosinophilic Granulomatosis With Polyangiitis (EGPA)

EGPA is associated with asthma, peripheral blood and tissue eosinophilia. This disease is classically considered as a Th2-driven inflammatory response with a key role of IL-5 in eosinophil recruitment (126). Activated eosinophils play a pathogenic role and secrete IL-17E/IL-25 that in turn enhance Th2 cytokine production. Patients with active EGPA have increased levels of IL-25 and tissue staining of nerve tissue specimens reveals the presence of IL-25 and IL-17RB^+^ T cells (127).

The frequency of circulating IL-17-producing CD4^+^ T cells is increased in active EGPA compared with inactive disease (128). In a pathologic colonic submucosa from an EGPA patient, there is a positive correlation between eosinophil count, crypt-to-crypt distance, the basement membrane-to-crypt distance with the percentage of Th17 cells (129). Finally, there is an inhibition of Treg differentiation (128, 130).

Results on AAV are summarized in **Figure 3C**.

#### 3.3.2 Immune-Complex-Mediated Vasculitis

##### 3.3.2.1 Anti-GBM (Anti-Glomerular Basement Membrane) Disease

Anti-GBM disease is an autoimmune disorder characterized by the presence of anti-GBM auto-antibodies bound to basement membrane in glomerular and pulmonary alveolar capillaries. It explains the rapidly progressive GN and alveolar hemorrhage that occur. The main target of auto-antibodies is the non-collagenous domain 1 of the α3 chain of type IV collagen (8, 131).

Many results came from different animal models (102). Mice injected with Th17 cells exhibit a neutrophil signature with increased neutrophil infiltration (132). The Th17 pathway allows the recruitment of destructive neutrophils through the expression of CXCL5 by kidney tubular cells which contribute to renal tissue injury (133, 134). Moreover, the Th17 subset induces GN with crescent formation and antigen-specific Th17 cells are the main contributors to renal tissue injury (135). Additional studies confirmed the pathogenic role of Th17 cells in anti-GBM disease (136–138). The Th17/Th1/Treg balance also plays a role in its pathogenesis (134, 139).

##### 3.3.2.2 Cryoglobulinemic Vasculitis

Cryoglobulinemic vasculitis is characterized by cryoglobulin immune deposits in small vessels that can affect skin, glomeruli and peripheral nerves (8). To our knowledge, there are currently no report on IL-17 involvement in vasculitis due to cryoglobulins. Results from a paper on mixed cryoglobulinemia associated with chronic hepatitis C virus (HCV) show an elevation of IL-17-inducing cytokines in patients with chronic HCV and mixed-cryoglobulinemia compared with HCV patients without vasculitis and healthy controls (140). This may suggest IL-17 involvement in this disease.

##### 3.3.2.3 Immunoglobulin A (IgA) Vasculitis (IgAV)

IgAV is a vasculitis more common in children characterized by IgA1 deposits affecting small vessels. Clinical symptoms include cutaneous purpura, arthralgias and/or arthritis, acute enteritis and glomerulonephritis (8, 141).

Immunostaining of renal biopsies shows IL-17 expression in all specimens studied with IL-17^+^CD3^-^CD4^-^ cells in the tubules and glomeruli, and IL-17^+^CD3^+^CD4^+^cells in the interstitium. Compared to control patients, glomerular and tubular grades of IL-17 expression are higher and IL-17 expression is correlated with proteinuria (142). Tubular cells may be an extra-immune source of IL-17 when triggered with injury (143). Moreover, the proportion of circulating Th17 cells and serum IL-17A level are increased in IgAV children compared with healthy individuals. Once again, the Th17/Treg imbalance may play a role in IgAV pathogenesis and is correlated with disease activity (144–146).

##### 3.3.2.4 Hypocomplementemic Urticarial Vasculitis HUV (Anti-C1q Vasculitis)

HUV is a leukocytoclastic immune complex vasculitis accompanied by urticaria and hypocomplementemia with anti-C1q antibodies. This disease can induce GN, arthritis, pulmonary disorder and eye inflammation (147). To our knowledge, no paper reports results on Th17 involvement in HUV.

### 3.4 Variable Vessel Vasculitis (VVV) – Behçet’s Disease (BD)

VVV can affect vessels of any size and of any type. Behçet’s disease end Cogan’s syndrome are the two entities included in CHCC2012 (8).

BD often refers to the Silk Route disease because of its prevalence in the Middle-East and far-east Asia. Skin and mucosa lesions are the most common clinical manifestations but the prognosis mainly relies on vascular and neurological involvement (148). BD is characterized by thrombosis, aneurysms and occlusions (149).

The frequency of circulating Th17 cells and serum levels of IL-17A are increased in BD compared with healthy controls and are correlated with disease activity. Circulating Treg proportion is decreased in BD and the Th17/Treg ratio is higher in BD patients compared with controls (150–153). The Th17 and Th1 hyperactivation observed in BD is partially mediated by a decreased in B and T lymphocyte attenuator (BTLA) expression (154). Serum amyloid A promotes Th17 differentiation in BD (155). Immunostaining reveals that IL-17^+^cells infiltrate the erythema nodosum-like eruption in the skin of BD patients. IL-17A producing cells are found in the cerebrospinal fluid, in brain parenchyma inflammatory infiltrates and in intracerebral blood vessels from patients with active disease (156). Finally, IL-17A and IFNγ production are associated with enhanced innate immune response, early neutrophil tissue infiltration and late adaptive immunity (149, 157, 158). Results concerning BD pathogenesis are presented in **Figure 3D**.

## 4 Targeting IL-17 and Th17 Cells in Vascular Inflammation

Almost all studies described above only show an increased level of IL-17 and/or of Th17 cells but results on their real pathogenic roles are limited. IL-17A acts as a primer, or a consort, in a complex network of cytokines and is looking for synergy, with the typical example of TNFα. Lessons from RA and multiple sclerosis, where IL-17 inhibitors do not work as expected, must be learnt when considering new therapeutic strategies for vasculitis (17, 159). The targeting of IL-17 alone may not be sufficient to control these diseases and combined inhibition should be considered. To potentiate an eventual benefit from targeting this cytokine, the identification of patients with bioactive IL-17A would be of interest (17). Nonetheless, targeting IL-17 pathway could be part of new strategies to control vasculitis and methods to target it are firstly described. Then, approved biologics and on-going clinical trials are presented. Finally, other treatments to inhibit IL-17 are detailed.

### 4.1 Tools to Target the IL-17 Pathway

#### 4.1.1 Direct Modulation of the IL-17 Pathway

Two antibodies are now available for targeting directly IL-17A (sekukinumab and ixekizumab) and constitute the more straightforward option. Recently, bi-specific antibody directed against both IL-17A and IL-17F (bimekizumab) was tested in psoriasis and may be more effective than secukinumab, which only inhibits IL-17A (160). These results were expected as IL-17A and IL-17F act synergistically (27). In the same vein, bispecific antibodies that block TNFα and IL-17A are currently developing. Finally, the targeting of IL-17RA with brodalumab and the inhibition of RORc constitute alternative strategies (6, 35).

#### 4.1.2 Indirect Inhibition of the IL-17 Pathway

**Figure 4:**
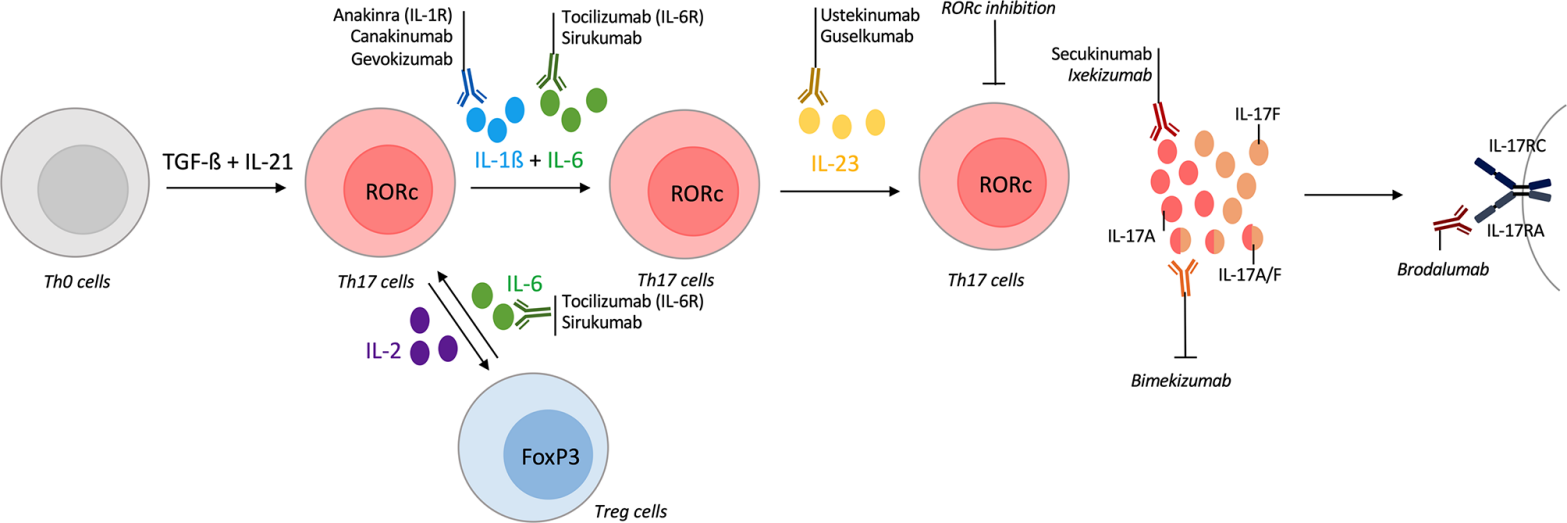
Tools for targeting the IL-17/Th17 pathway in vasculitis. T-helper (Th) 17 differentiation is a multi-step process with different cytokines involved. Their inhibitions could act on Th17 differentiation and modify the course of vasculitis. The first step of Th17 differentiation is mediated by interleukin (IL)-1ß and IL-6. Inhibitors of IL-1ß (canakinumab, gevokizumab) or IL-1 receptor antagonist (anakinra) are available and some were tested in vasculitis. Inhibitors of IL-6 (sirukumab) or of its receptor (tocilizumab) were approved in selected giant-cell arteritis (GCA) patients. IL-6 inhibition also acts on the Th17/regulatory T cells (Treg) dysregulation that occurs in vasculitis. Another way to modulate the Th17/Treg balance is to use low-dose of IL-2. Receptor-related orphan receptor (RORc) inhibition also suppresses Th17 lineage. Then, IL-23 is required for Th17 differentiation and ustekinumab is an inhibitor of the IL-12/IL-23 axis, guselkumab specifically targets IL-23. Promising results in Behçet disease (BD) are described. After their differentiation, Th17 cells produce IL-17A which could be inhibited by secukinumab or ixekizumab. Bispecific antibodies against IL-17A/IL-17F are currently developing with bimekizumab. brodalumab is an IL-17 receptor antagonist. All biologics targeting directly the IL-17 pathway are represented but secukinumab was the only one tested in vasculitis.

Th17 differentiation is a multi-step and a dynamic process. Cytokines required to Th17 differentiation include IL-1ß, IL-6 and IL-23. In addition to these cytokines, low dose of IL-2 allows a shift toward Treg cells at the expense of Th17 population (15, 161). Conversely, targeting of IL-6 receptor in GCA increases Treg population and reverts their pathogenic phenotype observed during active disease (38). Regarding these elements, the inhibition of a cytokine involved in these processes is a way to interfere with the Th17 pathway. Many biologics are available to target these cytokines but only the ones tested in vasculitis are described here (**Figure 4**). Statins and metformin, that are widely used, are also described, as *in vitro* inhibitors of the IL-17 pathway.

### 4.2 Approved Biologics in Vasculitis and On-Going Clinical Trials

Biologics that have been tested in vasculitis are described with the idea that the inhibition of the Th17 pathway could modulate disease’s activity. However, the cytokine itself that is inhibited could also play a role in the pathogenesis. The discussion below only provides some elements for thought.

#### 4.2.1 Inhibition of IL-17

To date, very few trials have been conducted in vasculitis. A trial is on hold with secukinumab in patients naïve to biologics and with newly diagnosed or relapsing GCA (162).

Some results suggest that secukinumab could be efficient in BD as promising results come from patients with non-infectious uveitis (163). Trials to modify the Th17/Treg balance have been performed with low dose IL-2 administration and even more are on-going (NCT01988506, NCT04065672, NCT04387942) (161).

#### 4.2.2 Inhibition of IL-6

The inhibition of IL-6 in vasculitis could have a therapeutic effect in different manner, notably in an IL-17 dependent fashion. First, IL-6 is required for Th17 differentiation. This cytokine also plays a role in the Th17/Treg imbalance and IL-17A up-regulates IL-6 expression through NFκB pathway (1, 15, 16).

Tocilizumab is an IL-6 receptor inhibitor that was firstly used in RA (164). As IL-6 pathway is absolutely central in GCA pathogenesis, its targeting through tocilizumab was firstly tested (75). Tolicizumab was shown to restore a better Treg function than glucocorticoids (37). Trials were conducted in GCA and allowed for reductions in steroid doses and maintain remission (165, 166). Recent EULAR guidelines recommend the use of Tocilizumab as adjunctive therapy in selected GCA patients (14). Sirukumab efficacy, an IL-6 production inhibitor, is currently testing in GCA (167).

Tocilizumab can be considered in relapsing or refractory TAK when conventional disease modifying anti-rheumatic drugs (DMARDs) or TNF blockers are not sufficient to control disease activity (14, 168–170). Apart from LVV, Tocilizumab was also tested in BD with promising results and clinical trials are on-going (NCT03554161) (171, 172). In AAV, some case reports have been published but larger clinical trial are required (173).

#### 4.2.3 Inhibition of IL-1ß

IL-1ß is required for Th17 differentiation and enhances Th17 cell differentiation, as shown in BD (174). Different tools are available to block this pathway, either with IL-1 receptor antagonist (anakinra) or by targeting directly the cytokine itself (canakinumab, gevokizumab) (175). Clinical trials are on-going to test anakinra in addition to corticosteroids in GCA (NCT02902731). Anakinra is currently testing in KD (NCT02179853, NCT02390596). In BD, canakinumab (NCT02756650), anakinra and gevokizumab were tested and results suggest a mixed effect depending on clinical manifestations (176–181). Canakinumab was also tried in HUV and results are expected (NCT01170936).

#### 4.2.4 Inhibition of IL-23

As mentioned above, IL-23 is required for Th17 differentiation (15). Different biologics were developed to target this cytokine with the example of ustekinumab, that targets p40 subunit and thus potentially inhibits both Th1 and Th17 pathways (182). Precisely, ustekinumab was tested in a GCA patient and inhibited Th1 and Th17 polarization (183). A recent trial showed disappointing results but they must be analyzed carefully for several reasons (uncontrolled trial, time of analysis) (184, 185). Other trials displayed promising results and this treatment is currently testing in relapse or refractory GCA (NCT03711448) (186). Among LVV, ustekinumab was also tested in three TAK patients with promising results (187–189). Recently, guselkumab, that specifically binds to the p19 subunit of IL-23, is currently tested in GCA (NCT04633447). Concerning VVV, ustekinumab was tried in BD; at week 12, 70% of patients fulfilled criteria for complete response. As compared to baseline values, IL-17A levels were significantly decreased at week 12 (190). These observations were subsequently confirmed but results from other trials are expected (NCT02648581) (191).

### 4.3 Other Treatments to Target the IL-17 Pathway

Outside biologics, some treatments currently used in cardiovascular prevention and in diabetes can target the IL-17 pathway. For instance, statins are shown to reduce the pro-inflammatory and pro-thrombotic effects of IL-17A and TNFα on EC (192).

Indirect evidence of metformin’s effect on the IL-17 pathway comes from cancer. Metformin is an agonist of Sirtuin-1 whose activation reduces Th17 frequency in patients (193). A Chinese trial tested metformin in thirty BD patients. In terms of clinical manifestations, the overall favorable response rate was almost 90% and partial remission was obtained in the rest of patients. Inflammatory parameters were lowered by the treatment. FoxP3 and TGF-ß protein levels were increased while IL-17 expression was lowered suggesting that metformin mediates the Th17/Treg imbalance (194).

Other treatments used in RA or in chronic inflammatory diseases were shown to decrease Th17 response, as glucocorticoids suppress Th17 responses in GCA patients (77). JAK inhibitors were tested in TAK patients with a response in two of three patients treated with a decrease in Th17 cells and an increase of Treg ones (70). Recently, tofacitinib (TOF), which preferentially inhibits JAK1 and JAK3, was compared with methotrexate (MTX) in TAK. TOF and MTX were associated with glucocorticoids, the study included 53 patients (26 in MTX group, 27 in TOF group) and showed the advantages of TOF regarding complete remission induction, the prevention of relapse and the tapering of steroid dose compared with MTX. No serious side effects were observed in the TOF group during 12 months of treatment but results must be confirmed in larger cohort with longer follow-up (195). Similarly, baricitinib, which inhibits JAK1/JAK2, has been tested in GCA. Fifteen relapsing GCA patients were enrolled in an open-label pilot study and preliminary results demonstrated evidence of both efficacy and safety. Discontinuation of glucocorticoids was allowed in the majority of patients with relapsing GCA but larger trials are needed to confirm these results (196). Finally, the inhibition of the complement pathway with avacopan (anti-C5a receptor) in AAV showed promising results but its effects on the Th17 pathway have to be characterized (197).

Overall, more clinical data are needed to conclude on the potential benefit to specifically target IL-17 in these diseases.

## 5 Conclusion

It is now well established that IL-17A is involved in autoimmunity and in chronic inflammation. This demonstration came from both *in vitro* and *in vivo* experiments and was confirmed by the efficacy of IL-17 inhibitors in various articular and cutaneous diseases. The effects of IL-17A on the CV system were described locally and then at a systemic level. Interacting in a complex network of cytokines, IL-17A promotes inflammation, thrombosis and coagulation. Regarding these effects, results suggest that this cytokine is involved in various diseases ranging from atherosclerosis to vasculitis. Vasculitis treatment faces challenges as steroids constitute the cornerstone of care. The indirect modulation of the Th17 pathway through different biologics has shown efficacy, particularly in LVV with tocilizumab which is now recommended in selected cases. As an extension, IL-17A could constitute an attractive target in inflammatory CV diseases if the cytokine is considered in its complex network. Disappointing expectations from the use of IL-17 inhibitors in RA and in multiple sclerosis must be considered before going further into their application in vasculitis where the Th17 pathway may act as a culprit and a consort. Given that, the thoughtful use of these drugs could be of great benefit to patients.

## Author Contributions

MR: writing and figures. PM and AH: concept and proof reading. All authors contributed to the article and approved the submitted version.

## Funding

MR is supported by the Ecole de l’Inserm Liliane Bettencourt Programme. PM is a senior member of the Institut Universitaire de France. His laboratory is supported in part by the IHU OPERA.

## Conflict of Interest

PM holds a patent on the determination of bioactive IL-17A.

The remaining authors declare that the research was conducted in the absence of any commercial or financial relationships that could be construed as a potential conflict of interest.

## Publisher’s Note

All claims expressed in this article are solely those of the authors and do not necessarily represent those of their affiliated organizations, or those of the publisher, the editors and the reviewers. Any product that may be evaluated in this article, or claim that may be made by its manufacturer, is not guaranteed or endorsed by the publisher.
